# Portrait of a Geothermal Spring, Hunter’s Hot Springs, Oregon

**DOI:** 10.3390/life5010332

**Published:** 2015-01-27

**Authors:** Richard W. Castenholz

**Affiliations:** Institute of Ecology and Evolution, University of Oregon, Eugene, OR 97403, USA; E-Mail: rcasten@uoregon.edu; Tel.: 1-541-346-4530; Fax: 1-541-346-2364

**Keywords:** hot springs, cyanobacteria, chloroflexi, ostracods, thermophiles, photosynthesis, eastern Oregon

## Abstract

Although alkaline Hunter’s Hot Springs in southeastern Oregon has been studied extensively for over 40 years, most of these studies and the subsequent publications were before the advent of molecular methods. However, there are many field observations and laboratory experiments that reveal the major aspects of the phototrophic species composition within various physical and chemical gradients of these springs. Relatively constant temperature boundaries demark the upper boundary of the unicellular cyanobacterium, *Synechococcus* at 73–74 °C (the world-wide upper limit for photosynthesis), and 68–70 °C the upper limit for *Chloroflexus.* The upper limit for the cover of the filamentous cyanobacterium, *Geitlerinema (Oscillatoria)* is at 54–55 °C, and the *in situ* lower limit at 47–48 °C for all three of these phototrophs due to the upper temperature limit for the grazing ostracod, *Thermopsis.* The *in situ* upper limit for the cyanobacteria *Pleurocapsa* and *Calothrix* is at ~47–48 °C, which are more grazer-resistant and grazer dependent. All of these demarcations are easily visible in the field. In addition, there is a biosulfide production in some sections of the springs that have a large impact on the microbiology. Most of the temperature and chemical limits have been explained by field and laboratory experiments.

## 1. Introduction

Hunter’s Hot Springs, alkaline springs, with several sources in 16 ha (42.44088 °N, 120.36907 °W) are located 3.2 km north of Lakeview, Oregon (in the Goose Lake Valley of the Great Basin). They were discovered by westerners in 1832 by trappers of the Hudson’s Bay Company, and are now officially (United States Geological Survey) known as Geyser Hunter’s Hot Springs. They are privately owned as a commercial property, and, at present, the water level varies because of geothermal drilling operations. This operation and other alterations by recent human activity occurred after the studies reported here. There was a man-made geyser (“Old Perpetual”) that erupted every 40–120 s to 15–18 m at 93 °C until a few years ago. Several sources at Hunter’s Hot Springs issued at temperatures of ~93 °C, which at the elevation of 1448 m is less than 2 °C below the boiling point of pure water (94.8 °C). The heat, in general, is a result of indirect contact with the magma as well as by the friction created during shearing of the current faulting of the tilted fault block of the Abert Rim. The shear zone provides conduit for superheated water to escape upward from considerable depths through the scoriaceous basalts of the subsurface lava flows. The substrate into which the springs emerge is the lakebed of a pluvial (Pleistocene) lake, the remnant of which still exists as Goose Lake in a graben about 10+ km to the south of Lakeview. The soft and unconsolidated sediment of the springs consists of a large deposit of empty lucustrine diatom frustules that can easily be mistaken microscopically for the presence of a viable diatom population which only exists below about 35 °C.

Several of the outlet streams continue to run to ambient temperature; others are pools with a temperature gradient from center to edge. There are several similar alkaline springs in the eastern Oregon region with somewhat similar chemistries and seemingly similar microbiota (e.g., Barry’s Hot Springs, South Harney Hot Springs; Mickey Hot Springs, Borax Lake Hot Springs, Alvord Hot Springs, and others to the east, and southeast in Oregon, northern California and Nevada [1].

The macro-chemistry of Hunter’s Hot Springs has been measured a few times. There are no visible precipitates except for thin siliceous deposits on hard materials that had fallen in the springs. The major inorganic solutes are sodium (~8.6 mM), chloride (~3.2 mM), silicate (~1.2 mM), bicarbonate (~1.7 mM), and sulfate (~2.8 mM) with a specific conductance of ~1.1 mS). The source pH varies between 8.2 and 8.3. This chemistry is characteristic of the other hot springs of southeastern Oregon [1,2]. In Hunter’s the primary sulfate level was unusually high.

Temperature and chemical gradients enabled the explicit investigation of taxon composition and environmental variation in hot springs at selected points in the gradients and determine the visually sharp boundaries among different phototrophs. The primary aim of this review is to establish the justification for the well-defined upper and lower limits of the macro-constituents of these springs. These are relatively simple microbial ecosystems (compared with the taxon composition of most soils), thereby enabling detailed interrogation of the interactions between organisms and the environment. Here, I will summarize what is known about the visually apparent diversity of phototrophic bacteria (primarily cyanobacteria) that are the primary producers in this ecosystem.in what was once a relatively undisturbed hot spring in southeastern Oregon.

This paper builds on about 40 years of periodic research by R.W. Castenholz, his students and colleagues, which has resulted in over twenty publications and made Hunter’s Hot Springs, one of the best-studied hot spring systems in the world outside of Yellowstone National Park. However, most of these studies were made prior to the use of new techniques involving gene sequencing.

## 2. The Synechococcus/Chloroflexus Zone

**Figure 1:**
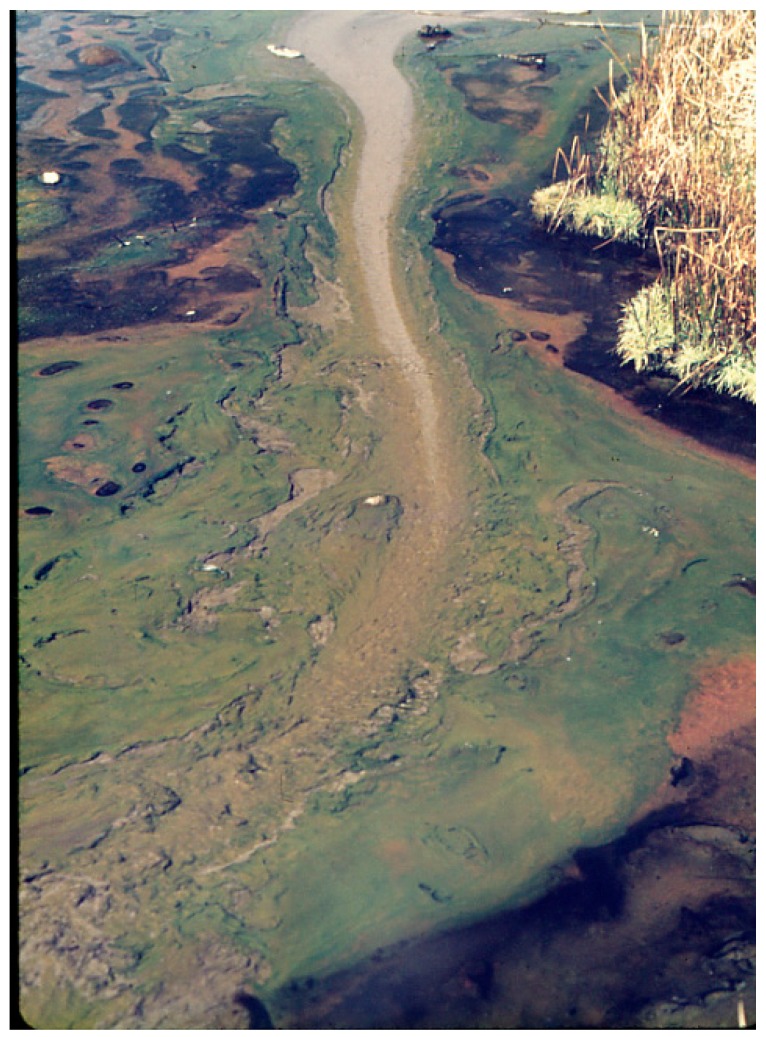
Hunter’s Springs from over ~93 °C at source (gray) to the green biofilm of *Synechococcus* (73–74 °C) that ends at 54–55 °C with the dark brown cover of *Geitlerinema (Oscillatoria) cf. terebriformis*, some of which has contracted to expose a salmon-colored undermat of *Chloroflexus* that had been hidden by the top-mat of the cyanobacterium, *G. terebriformis*.

**Figure 2:**
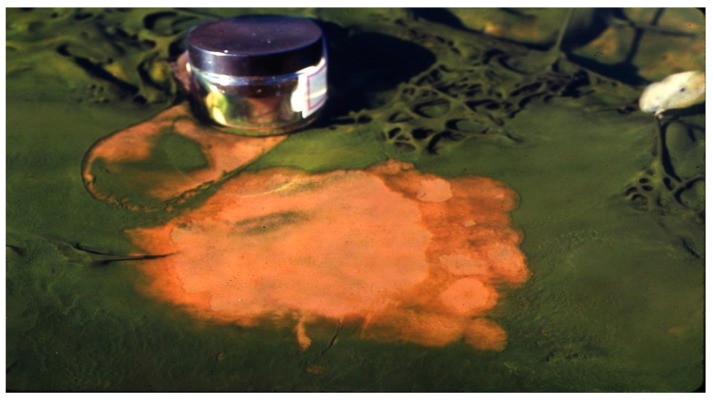
Hunter’s top mat of *Synechococcus* (green) with under-mat of *Chloroflexus* (yellow-orange) exposed in a thermal pool at 61–64 °C by blowing off the *Synechococcus* with a stream of water ejected from a syringe.

**Figure 3:**
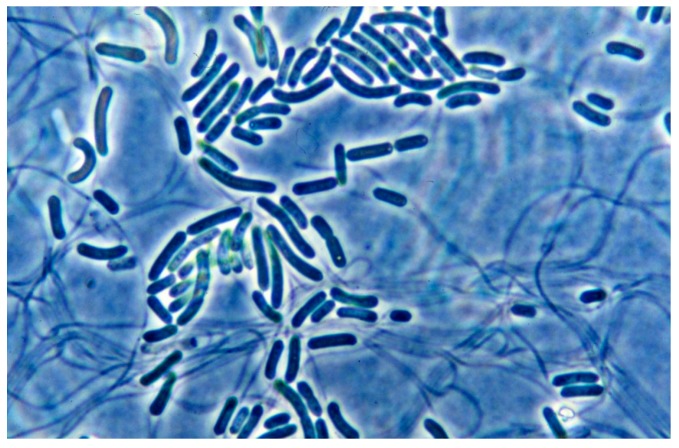
Photomicrograph of co-culture of *Synechococcus* (rod-like cells, 1.2 µm wide), some dividing, and *Chloroflexus aurantiacus* trichomes (~0.5 µm wide) in D medium at 45 °C.

The 93 °C water of some sources and water flowing downstream above 74 °C at Hunter’s presumably harbored non-photosynthetic Bacteria and Archaea, but these have not been identified. These clear water stream outpourings were generally not more than 1–3 cm in depth, although thermal pools were sometimes over a meter deep at the central source. At a mean temperature of 73–74 °C, a green edge of a biofilm or cover of *Synechococcus* sp. appeared and continued to about 55 °C, although below about 68–70 °C, a somewhat thicker mat with members of the Chloroflexi (e.g., *Chloroflexus* sp.) developed below, with the *Synechococcus* biofilm on top (Figure 1 and Figure 2). The green cover consisted of one or more species or strains of unicellular, rod-shaped, photoautotrophic *Synechococcus* to 55 °C (Figure 3). With decreases in the temperature gradient at least four thermotypes with identical morphology occurred [3,4]. This was confirmed by a later study that again recognized four or more thermotypes, each isolate with a distinctive growth rate measured over its complete temperature range [4,5]. These results, placed in phylogenetic context indicated that the highest temperature strain (University of Oregon, Culture Collection of Microorganisms from Extreme Environments strain No. 5245; OH 28) capable of growth to >70 °C, was ancestrally derived from lower temperature strains [5]. Additional information on the evolution of thermal stable Ribulose Bisphosphate Carboxylase/Oxygenase of the highest temperature and lower temperature strains has been provided [6,7]. Lower temperature strains grew with higher maximum growth rates than the high temperature type. In short, the highest temperature strain traded off a lower growth rate maximum and inability to grow at lower temperatures (<55 °C) with a higher temperature range. A similar and probably identical high temperature strain isolated from Hunter’s was characterized earlier by Meeks and Castenholz [8,9,10], who showed that this strain was an obligate thermophile with no net growth below about 55 °C. All of the *Synechococcus* strains, in field or culture, contain c-phycocyanin, allophycocyanin and chlorophyll *a* as the light-harvesting pigments.

The orange undermat of *Chloroflexu*s, was embedded in a “gelatinous” matrix, which was probably created as an extracellular polymeric substance of *Chloroflexus* and other microorganisms (Figure 3). It has been shown that in cyanobacterial mats essentially all of the radiation in the visible range (~400–700 nm) is absorbed by the cyanobacterial top layer of ~1–2 mm. It was shown in other studies that near infra-red radiation penetrated the top cover of *Synechococcus*, although at low intensities, but at sufficient levels to allow the *Chloroflexus* to operate as a photoheterotroph, using bacteriochlorophyll Bchl *c* (740 nm maximum) and Bchl*a* (802 and 865 nm) as major radiation-harvesting pigments [11]. The top portion of the under layer of Chloroflexi presumably obtains sufficient organic substrates produced by the overlying photoautotroph (e.g., glycolate, lipids, polyglucose with subsequent dark fermentations to acetate and propionate [4]. All of these products of *Synechoccocus* are available for photoheterotrophic or heterotrophic growth of Chloroflexi. Micro-stratification within the *Synechococcus* top layer in Yellowstone demonstrated a vertical differentiation into high light and lower light-adapted ecotypes [4,12]. Other *in vivo* studies of *Synechococcus* populations in Yellowstone hot springs (e.g., Mushroom and Octopus springs) included a demonstration of diel nitrogen-fixation and other more obvious metabolic processes that included night-time fermentation [13].

## 3. The Geitlerinema/Chloroflexus Zone

**Figure 4:**
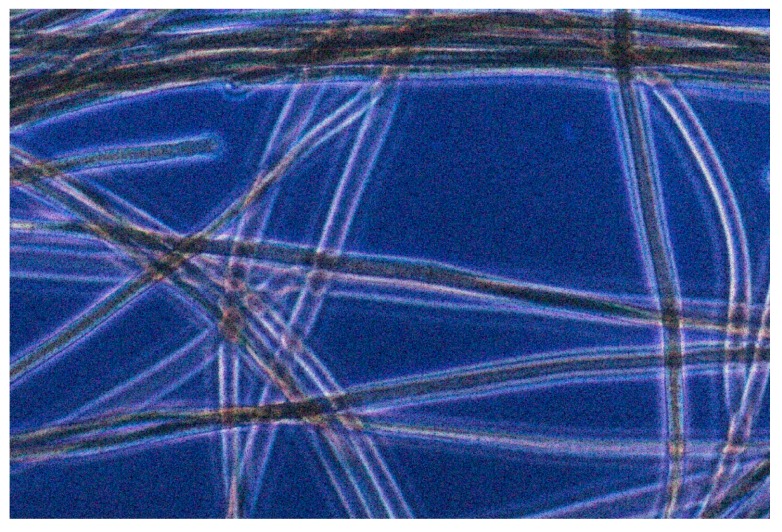
*Geitlerinema terebriformis* in culture (trichomes ~5 µm wide) in D medium at 45 °C.

**Figure 5:**
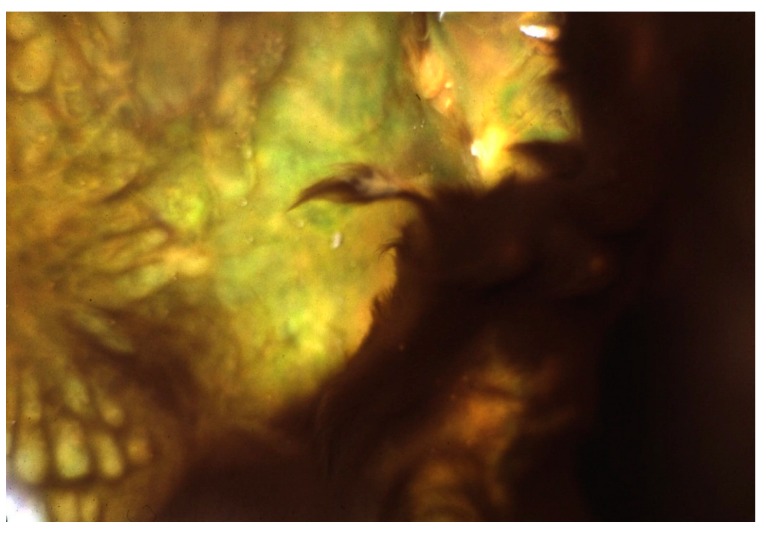
*Geitlerinema* *terebriformis* “mat” on right, with edge at ~55 °C, “riding over” *Synechococcus* topmat during low light period. The length of the view is ~6 cm (Figure 3 in [16]).

**Figure 6:**
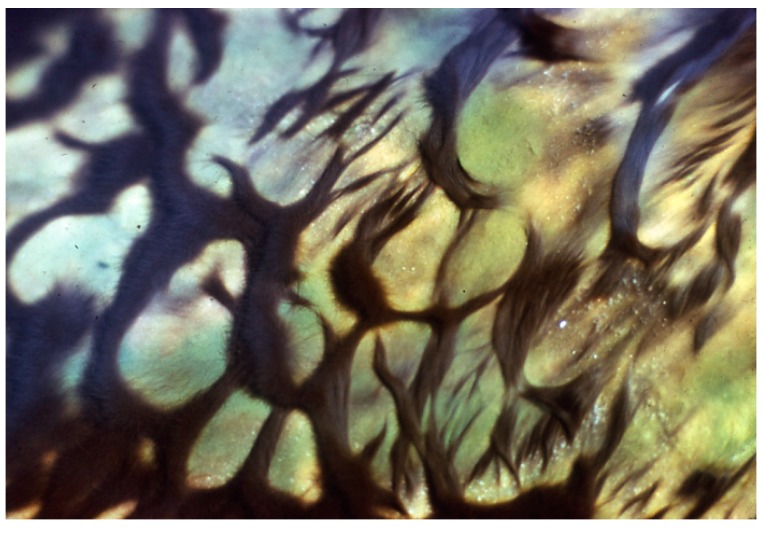
*Geitlerinema terebriformis* in contracted fascicles over *Synechococcus* topmat as a result of high, mid-day, summer light intensity. Temperature ~50 °C.

Although one the fastest growing isolates of *Synechococcus* (e.g., CCMEE strain No. 5241; OH 20) had its temperature for most rapid growth at about 45 °C [3,5], or strain 53 [3]. This temperature zone of the *Synechococcus* in the field did not reveal itself very often because of a dense cover of another cyanobacterium, *Geitlerinema (Oscillatoria)* *cf.* *terebriformis* (Figure 4). This motile oscillatorian formed a dark mat of intertwining filaments covering *Synechococcus* and all other phototrophs at 54–55 °C and below during most solar conditions (Figure 1 and Figure 5). This filamentous, motile cyanobacterium became the predominant phototroph up to about 54–55 °C (the highest limit and also nearly the maximum growth rate) [14]. This phenotype and 16S rDNA genotype is known from isolates from other hot springs in the eastern Oregon area, and even as distant as Saudi Arabia [4]. C-phycoerythrin is the primary light-harvesting pigment (545 nm maximum) giving the mat its dark color, although phycocyanin and allophycocyanin are present, as in almost all cyanobacteria. This cyanobacterial mat under low light was dense and continuous due to a mass of intertwining trichomes, and as a complete mat, and under low light (morning or afternoon in summer) absorbed up to 99% of visible radiation (Figure 5) and [15]. Normally, in response to high light intensity (e.g., after 9–10 am in summer and up to >1500 µE m^−2^ s^−1^ or 700 Wm^−2^) the motile trichomes of *G. terebriformis* responded in two ways. If the *G. terebriformis* mat rested on top of a rather hard “gelatinous” substrate, the main response was a mass clumping of trichomes into dense “balls” or “fascicles” (Figure 6). The rapidity of this response is temperature and light intensity dependent, e.g., [16,17,18]. Before 1967 the ecological advantage of this response was not obvious. It is now realized that the escape from high light inhibition occurs with this “clumping” phenomenon, since there is a continuous gliding motility of trichomes in contact with each other within the clump or fascicle so that no single one is exposed to high light for more that a few minutes or seconds. Since gliding motility requires a trichome rotation, the “tail-end” of a trichome is usually entangled with other trichomes, a compulsory contraction occurs. In culture, a dense, uniform suspension of trichomes in liquid medium spread in a 4.5 cm diameter glass petri plate contracts into a dense population of only 1 cm diameter in 130 s at 47 °C under ~40 Wm^−2^ irradiance, from coolwhite lamps, ~6% of highest outdoor intensity [16,17,18]. The rate of re-colonization of *G. terebriformis* on a denuded substrate in the field was ~1 cm hr^−1^ from the edge of an intact mat [16,17]. *G. terebriformis* trichomes can glide forward at ~0.5 mm^−1^ min under ideal temperature and light intensity [19].

**Figure 7:**
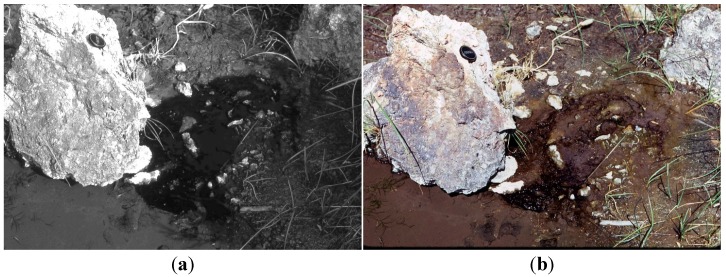
(**a**) *Geitlerinema terebriformis* “mat” in shade and 45 °C over sediment at 9:25 am, 27 July (Kahneeta Hot Springs) (Figure 4A in [15]); (**b**) Same “mat” at 10:00 (full sun in summer) with *G. terebriformis* largely absent due to downward vertical migration. Lower mat termination at meeting with cold water of river (Figure 4C in [15]).

**Figure 8:**
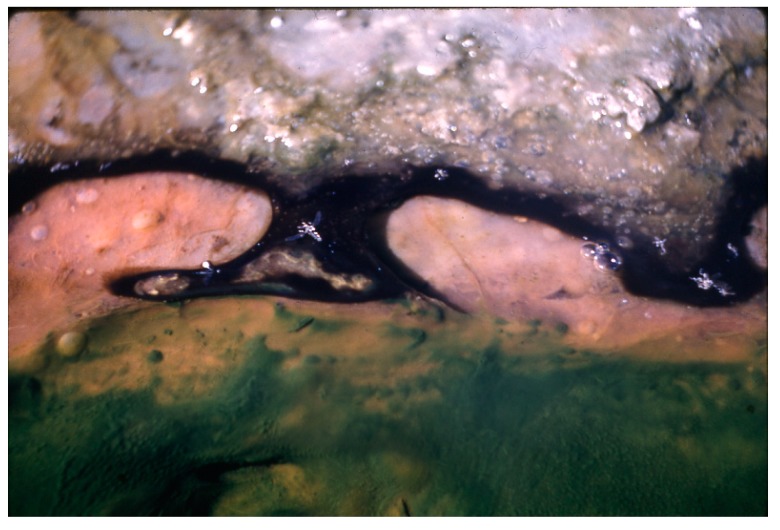
*Geitlerinema terebriformis* mat contracted and retreating under high midday light intensity at 49–53 °C, revealing *Chloroflexus* undermat, originally covered by the motile *G. terebriformis* mat during period of low light (Figure 7 in [15]).

Castenholz [15] showed that at <55 °C in this and similar hot springs (e.g., Kah-nee-ta Hot Springs, Oregon), when the substrate was soft, *G. terebriformis* trichomes had a vertical downward “escape” movement to 1–2 mm below the substrate under high light intensity in addition to the clumping response [15,16,18] (Figure 6 and Figure 7). In addition to the consolidation of the *G. terebriformis* mat in response to increasing light intensity, the mat edge showed a phobic response to a temperature above 54–55 °C, *i.e.*, upper temperature limit for growth when the stream flow changed [15]. The convergence and retreat of the mat with increasing light intensity or the advance of higher temperature also exposed, in many cases, an under-mat of orange or pink-colored *Chloroflexus* (Figure 8). This under-mat of *Chloroflexus* was visible as well at higher temperatures, below the *Synechococcus* top cover below about 68 °C (Figure 1 and Figure 2). *G. terebriformis* did not occur in at least one of the springs at Hunter’s (*i.e.*, “Fenceline” an unofficial name) [20]. No macro-chemical differences were shown for this spring, and the basis of this difference was never established.

In all other spring streams and pools at Hunter’s, *G. terebriformis* was present below about 55–54 °C, completely covering the lower temperature mat of *Synechococcus*, at various times of day, and this often resulted in an nearly absent or discolored cover of *Synechococcus*, a result of the nearly complete light shield by *G. terebriformis.*

## 4. The Ostracod/*Pleurocapsa/Calothrix* Zone

**Figure 9:**
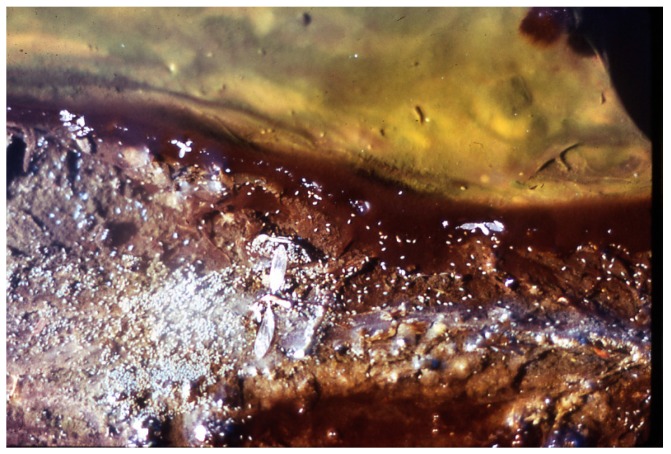
*Synechococcus* cover (>55 °C) with *Geitlerinema terebriformis* cover beginning at 55 °C and ending at about 48 °C due to grazing by the ostracod, *Thermopsis thermophile* (small white dots). (Figure 19.5 in [27]).

**Figure 10:**
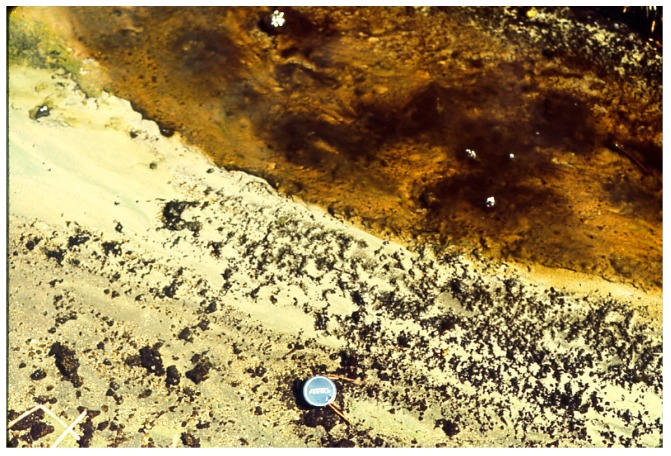
*Geitlerinema terebriformis* cover in Hunter’s stream that has been terminated at ~48 °C by ostracod grazing, revealing dark patches of *Pleurocapsa/Calothrix* in ostracod zone below 47–48 °C.

**Figure 11:**
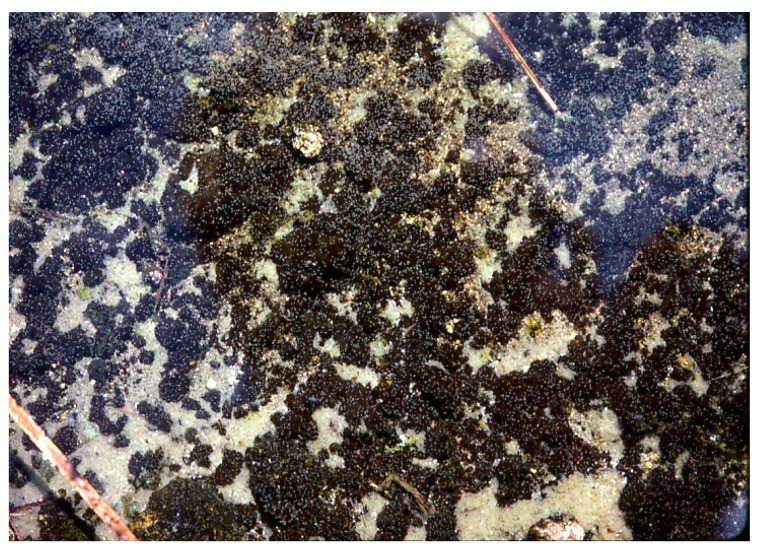
*Pleurocapsa*, leathery nodules with population of ostracods (white dots) over the area (horizontal length ~12 mm) in a Hunter’s stream at ~45 °C.

**Figure 12:**
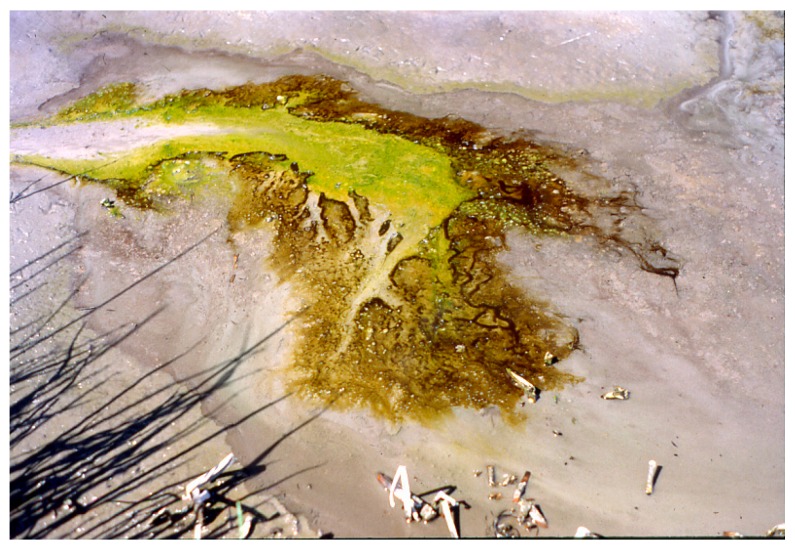
Newly established *Synechococcus/Geitlerinema terebriformis* population on soft gray substrate after 30+ days, but without the natural introduction of ostracods. In this case, the *G. terebriformis* cover gradually tapered out below about 35 °C much lower than 47–48 °C that would have been the temperature of truncation if ostracods were present.

**Figure 13:**
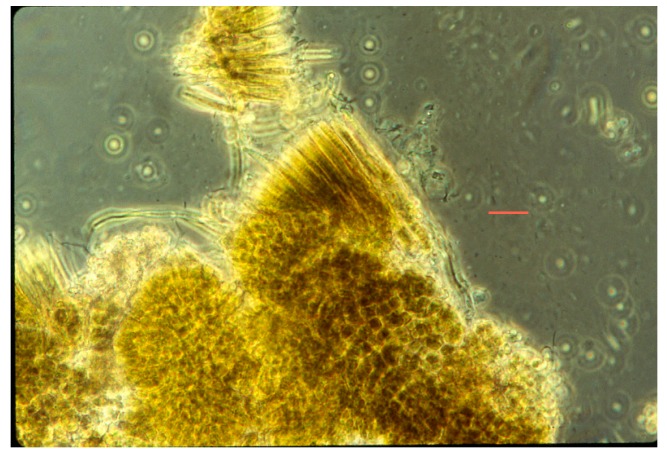
*Calothrix* (tapered filaments) extended from base of aggregated cells of *Pleurocapsa,* (non-filamentous); from piece of nodule from ~45 °C, scraped off hard substrate. Bar = 5 µm.

**Figure 14:**
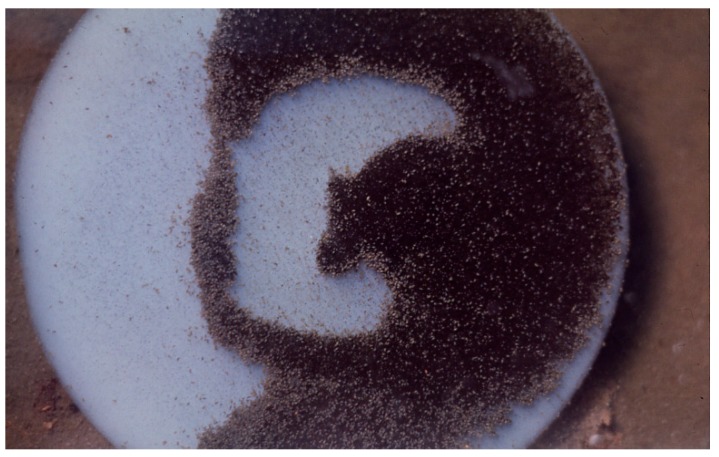
Agar nutrient slab on which *Pleurocapsa* had been grown uniformly in lab (in petri dish) and then placed in “Fenceline” Spring at Hunter’s at about 45 °C and allowed to be grazed upon by a natural ostracod population shown grazing here (small dark specks), after about 12 h with about 800–1000 animals cm^−2^, see [20].

Although *G. terebriformis* was quite capable of substantial growth below 47 °C [21], the dark phycoerythrin-containing mat was abruptly attenuated at about 47–48 °C, the upper temperature limit for the herbivorous, thermophilic ostracod (*Thermopsis thermophile*; formerly *Potamocypris* sp.) that was active and reproductively competent up to 47–48 °C [20,22,23]. *T. thermophile* and other species of ostracods have never been found in geothermal springs in Yellowstone where ephydrid flies and their larvae comprise the major grazers, but at lower temperatures (e.g., 43–44 °C). This micro-crustacean was very efficient in consuming *G. terebriformis* that appeared to be its “favorite food” in these springs. In laboratory feeding experiments they eliminated *G. terebriformis* very quickly with natural-populations [20,23] (Figure 9). However, the elimination of *G. terebriformis* below ~47 °C in springs and pools containing the ostracod, allowed and promoted the slow establishment of a nearly grazer-resistant population of a tough, leathery mat of nodules composed of *Pleurocapsa* sp. and *Calothrix* sp*.* Both contained the UV-shading pigment, scytonemin [24] (Figure 10 and Figure 11). In a few newly established springs at Hunter’s, no ostracods were present, in which case, the *G. terebriformis* mat extended to temperatures below about 30 °C, although it tapered out visibly with a lower suboptimal temperature below about 35 °C (Figure 12). Slow growth was demonstrated in the laboratory at temperatures as low as 28 °C [25]. The ostracod populations that were able to decimate the *G. terebriformis* cover and also ranged over the leathery nodules of *Pleurocapsa/Calothrix* averaging about 100 animals per cm^2^ in the 40–45 °C range in one stream (Figure 11), but often reached densities of over 2000 per cm^2^ when higher temperature water eddies circled and corralled the animals [20]. The grazer-resistant, grazer-dependent population of *Pleurocapsa* and *Calothrix* often consisted of a basal crust of *Pleurocapsa* that consisted of a tight aggregation of dark-colored, thick-walled cells with embedded upright-growing filaments of tapered *Calothrix* with basal heterocysts (Figure 13). It is almost certain that these heterocyst-containing filaments are capable of N_2_-fixation, and it is quite likely that the *Pleurocapsa* component was benefited by this process. It was observed that the upright *Calothrix* filaments grow as a “lawn”, extending above the crust of *Pleurocapsa*, but were usually truncated by the grazing of the ostracods, only to be extended again by later growth of the *Calothrix* filaments [26]. In this consortium the ostracods grazed on the extended tips of *Calothrix* (Figure 13). Most dividing cells of *Calothrix* are near the base of the filaments, close to the site of N_2_-fixation (*i.e.*, the heterocysts) therefore allowing continuous growth from below. The *Calothrix* never revealed colorless, terminal hairs, but it is unknown whether this was a result of high phosphate concentrations, which would inhibit the growth of these hairs, or a result of the effective grazing by the ostracods (Figure 13). This community occurred downstream (<~47 °C) in the chemical gradient where combined nitrogen is diminished due to the upstream use by mainly non-nitrogen-fixing cyanobacteria [20]. Recent findings have shown that some strains of *Synechococcus* in Yellowstone springs fix nitrogen, but only in late afternoon and early morning [4,13]. The existence or impact of this ability has not been investigated in Hunter’s springs. It was demonstrated in culture that the effect of grazing was considerably more effective and rapid in mono-cultures of *Calothrix* grown on agar nutrient discs, than on *Pleurocapsa* on agar discs, both immersed in springs at the appropriate temperature. This was expected, since the latter forms the basal, most resistant “leathery” crust in nature (Figure 14). In one spring (“Fenceline”) where the *G. terebriformis* cover was absent, the ostracods fed and truncated the *Synechococcus/Chloroflexus* mat at about 47 °C, the normal upper temperature limit for the animals [20]. However, these animals made feeding, swimming sorties to higher temperatures, but became comatose and closed their carapaces in a short time with increasing temperature. For example, forays to over 50 °C initiated this state often in 10–20 min [20,23]. Nevertheless, the stream current (from high to low temperature) carried comatose ostracods down to favorable temperatures (*i.e.*, below 47 °C) where swimming recovery generally occurred. In these eastern Oregon springs no other native grazers, besides ostracods, occurred, although an occasional tiger beetle was observed feeding on the ostracods, and an introduced population of “guppies” (*Poecilia reticulata*) thrived in some spring streams below 38 °C., and the bellies of these, upon examination, were full of ingested ostracods.

## 5. Sulfate Reducing Zone: Thermochromatium/Beggiatoa/Geitlerinema

**Figure 15:**
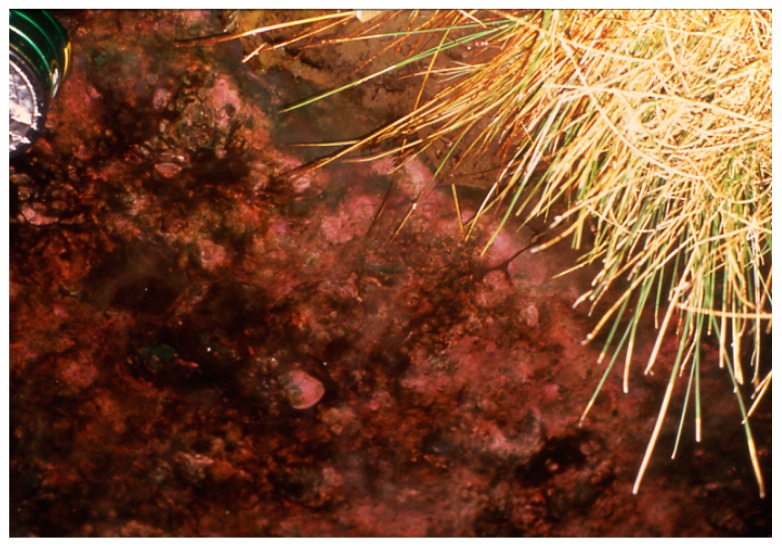
Portion of a southern Hunter’s stream at about 50 °C with high sulfide production, at night, showing *Thermochromatium cf.* *tepidum* (pink) at surface of soft substrate mixed with some dark areas of *G. terebriformis*.

**Figure 16:**
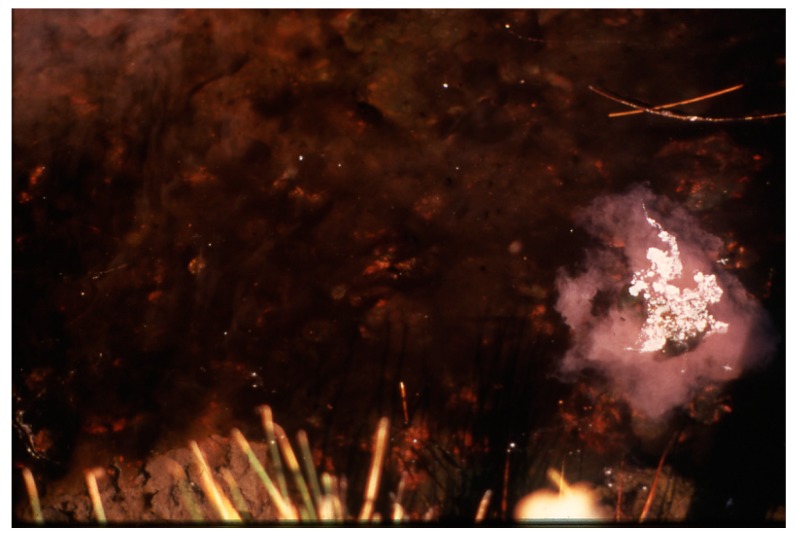
Swarm of swimming, ascending *Thermochromatium tepidum* (pink cloud at right) that had been covered by an opaque can for 2 h during 10 am on a cloudy day. In uncovered area, daytime cover of *Geitlerinema terebriformis* remained*.*

In some slow-flowing streams and warm pools (35–40 °C) in the southern part of the Hunter’s cluster of springs, sulfide concentrations (mainly HS^−^ at pH ~8.0–8.2) have been measured at 0.5 mM at the mat surface at night and up to 0.8 mM at 2–3 mm depth even in the daylight [18]. It was obvious that the high sulfide was a result of biogenic sulfate reduction, since soluble sulfide was absent in the inorganic source waters, and H_2_S arose from the mat at night with a strong odor [18]. In these pools and slow streams, at temperatures of ~50 °C and lower, a conspicuous under layer of purple sulfur bacteria (*i.e.*, *Thermochomatium* *cf.* *tepidum*) appeared at the top of the mat in darkness (Figure 15). Often, these flagellated cells swarmed at night (*en masse*) into the somewhat toxic water above, as pink clouds with cells containing “endocellular” grains of elemental sulfur (Figure 16). Although anoxygenic photosynthesis of the purple sulfur bacteria uses soluble sulfide as photo-reductant during daytime, at night the internal elemental sulfur of the cells gradually disappeared as the cells may have continued to grow, presumably using the internal S^0^ and O_2_ for aerobic chemo-litho-trophy [28].

**Figure 17:**
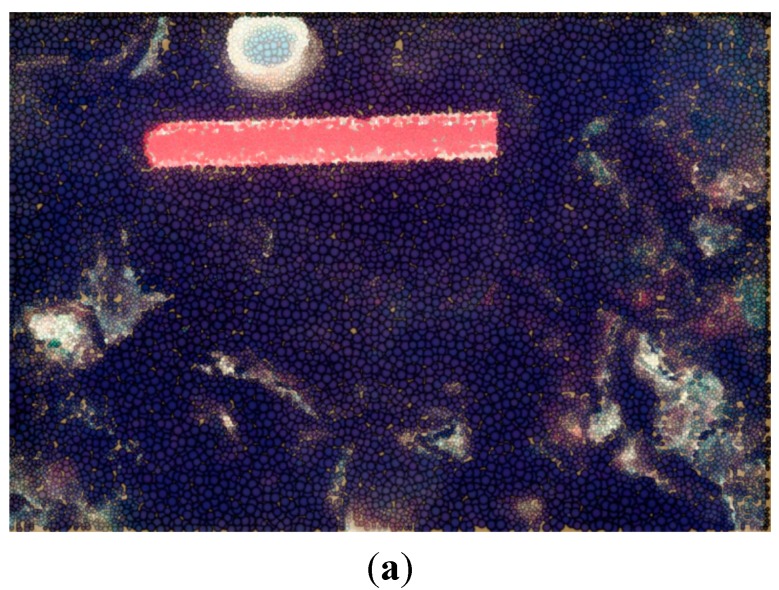

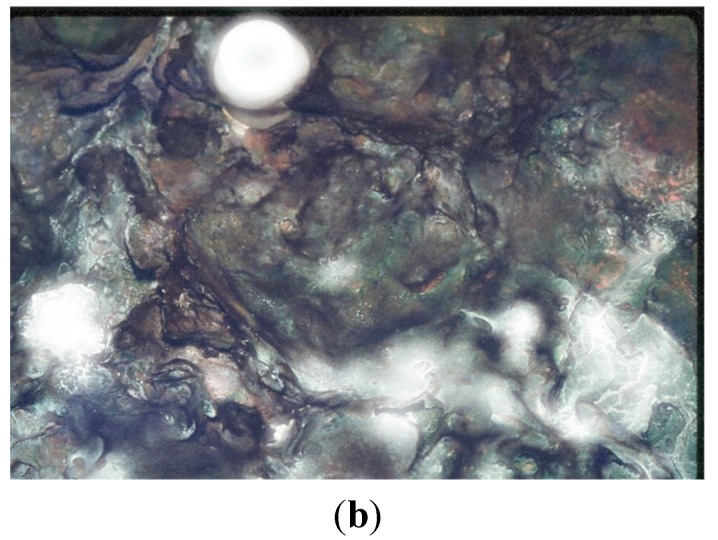
(**a**) Hunter’s pool at 9:25 am in April (~40 °C) with *Geitlerinema terebriformis*/*Oscillatoria princeps* cover (dark brown) but showing small patches of *Beggiatoa leptomitiformis* below the surface cover. Ostracods absent (Figure 2B in [18]); (**b**) Hunter’s pool at ~40 °C at 7:00 am (before sunrise, same day in April. *Beggiatoa* *leptomitiformis* (white) has migrated to the surface (complete darkness), with O_2_-sulfide interface 0.2–0.3 mm below surface of mat, and covering the *Geitlerinema terebriformis* below (Figure 2C in [18]).

Below ~40 °C, in many of the mats in pools, rather than streams, a species of sulfide-oxidizing, filamentous, non-photosynthetic bacterium occurred. The gliding trichomes of *Beggiatoa cf.* *leptomitiformis*, migrated upward within the mat at nighttime (Figure 17), Nelson and Castenholz [19]. Although this strain of *B. leptomitiformis* oxidized sulfide and deposited S^0^ “internally”, the benefit was apparently, that of sulfide detoxification [29], since this species is, in fact, a chemoheterotroph, in which acetate is a favored substrate [21]. Ostracods were absent in these pools, probably because of inhibition by the higher sulfide, therefore, allowing a complete *G. terebriformis* top cover of the mat in daytime, mixed with *Oscillatoria princeps* [30] and Figure 17a, but at night is partially covered by the upward moving *B. leptomitiformis* population (Figure 17b). It was shown that *B. leptomitiformis* motility is negatively affected by high light and that its lower position in the mat in daytime is a result of a negative step-up photo-phobic response [19]. At night there was an aerotactic movement toward the O_2_ at the surface of the mat [29], Figure 17b. The *G. terebriformis/O. princeps* population formed a cover in moderate light intensity in daytime. At night, in this scenario, the cyanobacteria remained in position, possibly because of the sulfide inhibition of gliding motility [18,31,32].

## 6. Discussion

**Figure 18:**
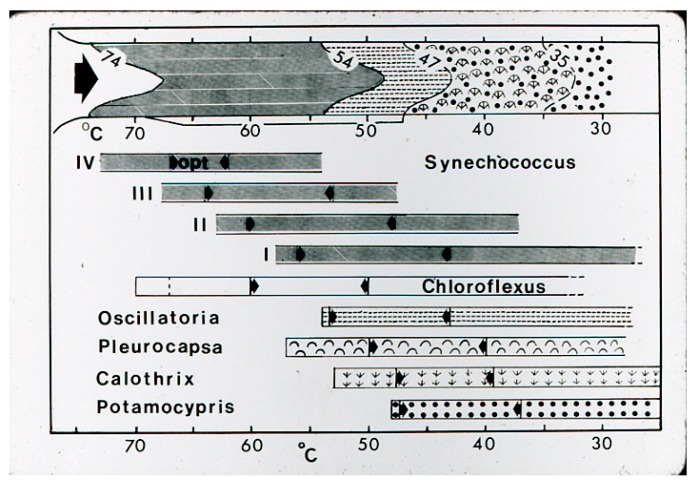
Cartoon summary of phototroph and ostracod distributions in Hunter’s streams. Upper portion with visible field distributions. Lower line below indicates *Chloroflexus* undermat distribution (47–68 °C) and ostracod, *Pleurocapsa, Calothrix* distributiions below 47 °C. The lower horizontal bars indicate ranges in culture of 4 *Synechococcus* thermotypes [3] and those of *Chloroflexus*, *Oscillatoria* (=*Geitlerinema*), *Pleurocapsa*, *Calothrix*, *and Potamocypris* (former name of the ostracod, *Thermopsis thermophile*)*.* The range between arrows indicates the approximate optimal range for growth in culture.

The positioning of all phototrophic microorganisms and the herbivorous ostracod in the thermal gradient of Hunter’s Hot Springs is shown in a composite cartoon (Figure 18). It has been shown that in some cases the upper limit of some species is determined by temperature (e.g., *Synechococcus* high temperature type, *Geitlerinema*, ostracods, *Thermochromatium*, and probably *Beggiatoa.* But the lower limit of the massive distribution of *Synechococcus* is limited by *Geitlerinema,* and that of *Geitlerinema* and *Synechococcus/Chloroflexus* by the ostracods. Although it is known that *Pleurocapsa* and *Calothrix* are capable of growth at higher than the field distributions [26] the impact of ostracod grazing determines the actual distribution. All of the organisms present in Hunter’s Hot Springs tapered out because of suboptimal temperatures at ambient or <30 °C, and other phototrophs were only occasionally present (e.g., phycoerythrin-containing *Nostoc*, and filamentous green algae).

Although light intensity and daylength change from summer to winter (~15:09 summer; ~9:15 winter), the temperatures of the sources of Hunter’s Hot Springs remained the same, *i.e.*, they were not influenced by the seasons. However, the zones of the phototrophs also remained more or less intact, most were retracted toward the water sources. This was particularly true for the *Synechococcus* and *Geitlerinema* zones that were able to recolonize or move upstream. However, the more stable gelatinous *Chloroflexus* mat was to some extent left behind at less than optimal temperature. The most conspicuous change was that the *Pleurocapsa/Calothrix* crust was left behind at a suboptimal temperature. The recolonization rate of this community was very slow. Therefore, it should be assumed that the photosynthetic and N_2_-fixation rates of these cyanobacteria were at a suboptimal temperature, although this was not determined. The ostracods were present in similar densities in winter, and, as usual, were limiting the *Geitlerinema* mat at about 47 °C. It is possible that the effect of grazing on the crust of *Pleurocapsa/Calothrix* was more effective in winter because of the assumed lower productivity of these cyanobacteria, but this too was never established.
